# “Rust stain”: a rare mark in firearm suicide—a case report and review of the literature

**DOI:** 10.1007/s00414-021-02607-x

**Published:** 2021-04-26

**Authors:** Luca Tomassini, Daniele Paolini, Anna Maria Manta, Edoardo Bottoni, Costantino Ciallella

**Affiliations:** Department of Anatomical, Histological, Forensic Medicine and Orthopedic Sciences, Section of Legal Medicine, University “Sapienza” Rome, Rome, Italy

**Keywords:** Firearm, Suicide, Rust stain, Rust spot, Perls Prussian Blue

## Abstract

Rust stains are marks left by firearms in case of prolonged contact with the cutaneous surfaces. These peculiar signs along with other well-documented findings can guide the medical examiner in the determination of the manner of death, especially in case of firearm suicide. This paper presents the case of a 33-year-old male soldier who committed suicide by using a short-barreled weapon, whose trigger remained in contact with the first finger of his right hand, leading to the formation of a rust stain that perfectly reproduced its design. The forensic examination of the scene, the external cadaveric inspection, and the autopsy are described. For the evaluation of the histological findings typical of rust spots, the authors decided to replicate the phenomenon in an experimental setting using porcine skin. In order to provide an exhaustive overview on the formation and the features of rust stains, a review of the forensic literature concerning this rare mark was performed.

## Introduction

Firearm suicides are common and well-described in the literature. However, they may represent a significant problem for the investigators, as in these cases, the determination of the manner of death can be challenging [1, 2]. Marks left by the gun on the person holding it, such as signs of contact-range gunshot, soot residues on the hands, and bloodstains on the weapon, may help the forensic pathologist to discriminate between suicide and homicide [1, 3–7]. Among the traces and injuries that a weapon can leave on the shooter, characteristic rust spots have been documented in the literature, especially in case of prolonged contact with the corpse. This peculiar type of stain remains imprinted on the skin and cannot be removed by wiping it [8, 9]. The case report presented describes a young soldier who committed suicide in the barracks by using a short-barreled weapon, which then remained in firm contact with the first finger of the right hand, causing the formation of a rust spot that faithfully reproduced the pattern engraved on the trigger. This article also offers a review of the forensic literature concerning this unusual phenomenon, which appeared to be scarce.

## Materials and methods

The present study focuses on a case of death by short-barreled firearm, which occurred in 2000 in Rome and filed as a suicide. The medical examiner in charge of the Rome Public Prosecutor’s Office carried out the inspection, the external examination, the autopsy, and the histological investigations; all phases were documented through photographic surveys. In order to study the rust spot imprinted by the weapon, this finding was photographed and subsequently compared to a Beretta pistol identical to the one found during the inspection. In addition to reporting all the elements inherent to the case, a review of the scientific literature on the phenomenon of rust spots from 1914 onwards was performed using the cross-entries “rust stain” and “suicide” on PubMed.

## Results

During the bibliographic research, a total of 14 articles on the phenomenon of rust stains were found, 6 of which were written in German language.

## Case report

**Fig. 1:**
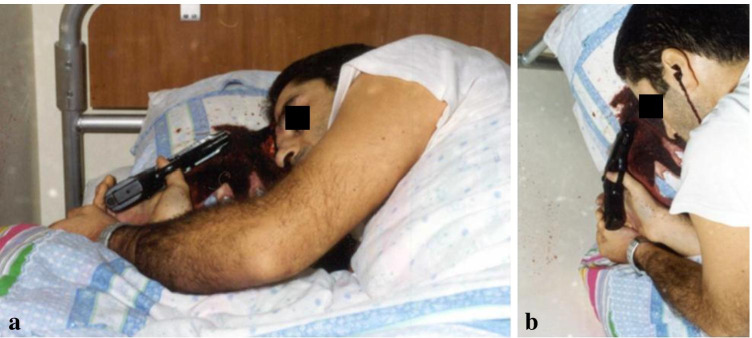
Position of the body at the time of medical examiner’s arrival. **a** Position of the body. **b** Position of the hands with respect to the weapon

**Fig. 2:**
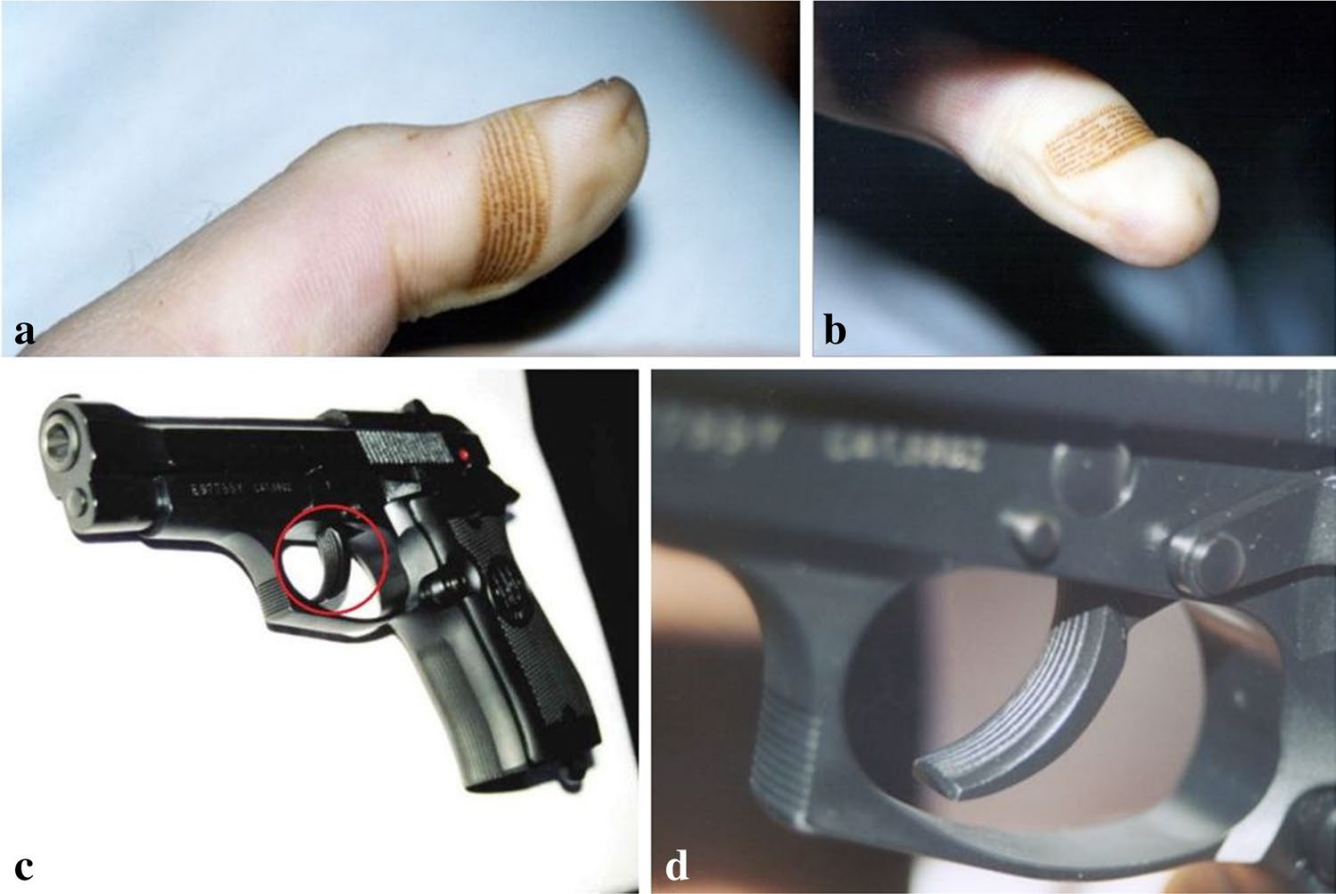
Comparison between the rust stain and the trigger (**a**–**b**). Rust stain located on the palmar surface of the first finger of the right hand (**c**–**d**). Detail of the trigger of the Beretta 84F—note the design of the trigger that perfectly coincides with the pattern of the rust stain

On the morning of December 4, 2000, a 33-year-old man was found unresponsive in his bed in the barracks of the Italian army, with the weapon still in his hand. The victim had been drafted into the military corps 10 years earlier with a physical and mental fitness assessment; a divorce had been going on for about 3 months. The body was lying on his right side, with the right arm bent and positioned under the chest. The right hand was hanging from the right edge of the bed. The right thumb was in contact with the trigger, while the second and third fingers were wrapped around the butt of the gun. The left hand was positioned on top of the last two fingers of the right hand, closing them into a fist, with the two palmar surfaces touching. The barrel of the gun was facing towards the frontal bone of the victim (Fig. 1). The pistol was a Pietro Beretta Mod. 84F cal. 9 corto supplied by the Italian armed forces. The Italian judicial authority ordered an autopsy of the body which was performed at the Department of Legal Medicine of the University of Rome “La Sapienza.” At the external inspection of the corpse, 1 cm to the left of the midline in the frontal region, a stellate entrance wound 1 cm in diameter with 6 V-shaped projections surrounded by an abrasion collar and a contusion ring was detected. The bullet path originating from this lesion extended deep into the cranial cavity, where a comminuted fracture of the vault was identified. Bilateral periorbital ecchymosis, otorrhagia, and rhinorrhagia were also present. A brown-orange stain, consisting of 6 discontinuous ribbon-like *striae*, seriate, and parallel to each other, of which the two lateral ones were greater in width compared to the central ones, was observed at the level of the fingertip of the first finger of the right hand. The rust spot was about 1 × 0.5 cm in dimension, with a transversal development with respect to the major axis of the finger (Fig. 2). It was not removable by rubbing.

### Autopsy

Upon skull examination, a round hole 1.2 cm in diameter was detected on the frontal bone, at the level of the entrance wound. The brain showed diffuse subdural and subarachnoid hemorrhage. The bullet path originated in the left frontal pole and continued without decussating at the midline, extending posteriorly at the level of the homolateral occipital pole. The left frontal, parietal, and occipital lobes were collapsed. Diffuse intraventricular hemorrhage was also observed. Multiple fractures of the cranial vault and the cranial base were detected. A bullet was found within the left posterior cranial fossa. The projectile was deformed along its major axis and had a concave ogive. No more wounds were present. Following the investigations, the forensic pathologist’s conclusion regarding the cause of death was deadly cranioencephalic trauma caused by a contact-range gunshot wound.

### Histological examination

For the histological examination, the skin within the rust spot was sampled and stained with Hematoxylin–Eosin and Perls Prussian Blue methods. The microscopic analysis of the specimens allowed the medical examiner to reject some of the first hypotheses, which were initially formulated in an attempt to explain the etiology of the imprint. No signs of powder tattooing, burn, or ecchymosis were detected. The Perls method confirmed the presence of iron deposits in the epithelium, extending from the most superficial up to the middle layers of the dermis.

**Fig. 3:**
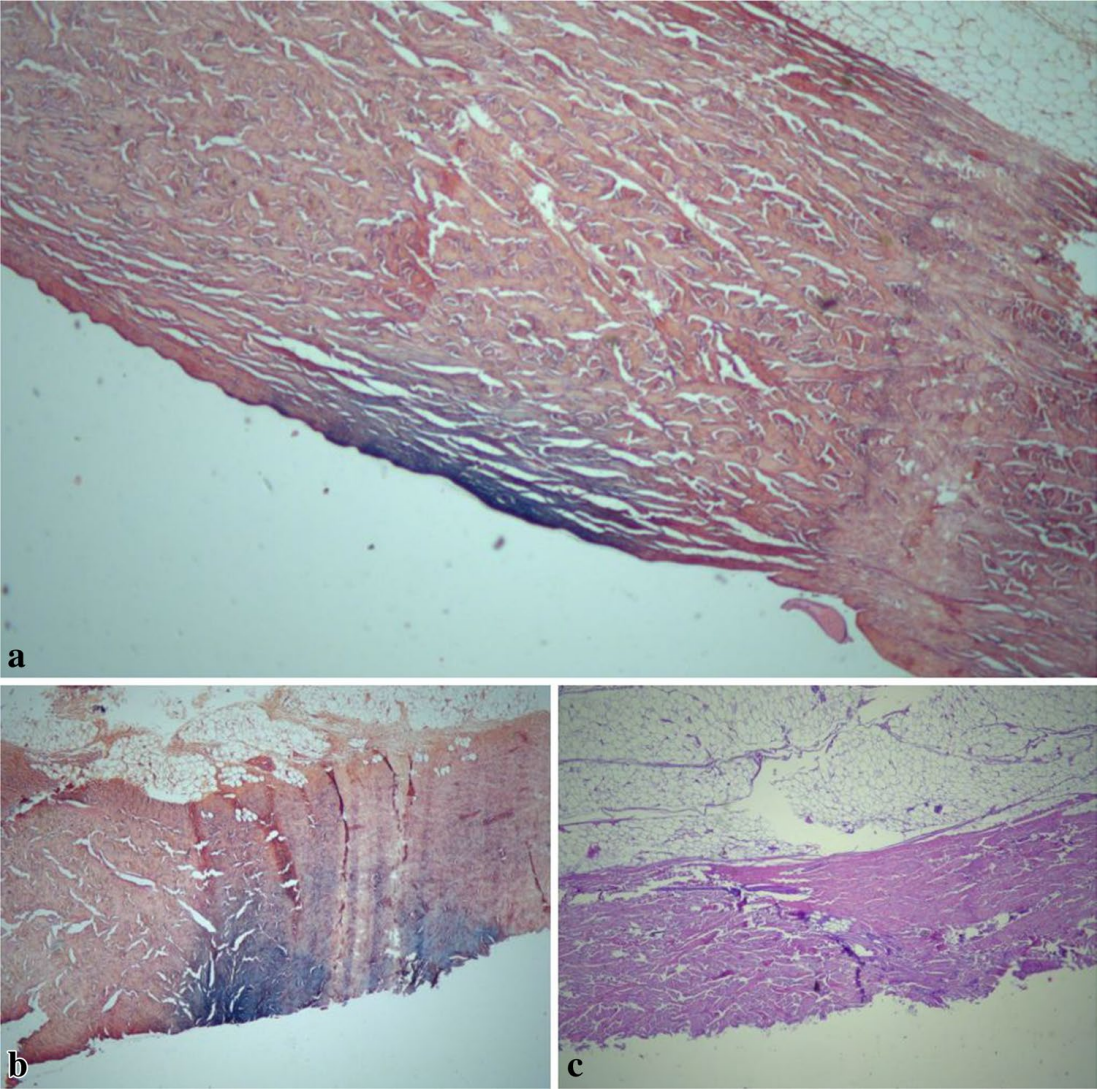
Histological section of porcine skin sampled in correspondence of macroscopically visible rust stain. **a**–**b** Perls Prussian Blue staining: bluish areas in the superficial layers; the dermis and the hypodermis can be recognized. **b** Hematoxylin–Eosin staining: absence of significant morphological alterations

In order to provide a thorough analysis and an exhaustive description of the microscopic features, the authors decided to reproduce the phenomenon of rust stains in an experimental setting using porcine skin, which is an adequate substitute for human skin, as it is structurally similar, readily available, and cheap. The procedure followed the main indications and methods described in Ulrich and Zollinger’s experiments [10]. An iron plate was positioned on top of the porcine skin with 1.5% NaCl solution. The sample was then left at a temperature of 4 °C from 7:00 p.m. until 8:30 a.m., to increase the probability of rust spot formation. At macroscopic examination, an orange-to-brown stain that could not be wiped off was observed. The skin was sampled, fixed in 10% formalin solution for 72 h, and then embedded in paraffin. The sections were stained with Hematoxylin and Eosin and Perls Prussian Blue dyes. Focal iron deposits were detected in the most superficial portions of the dermis: these appeared as blue spots among the surrounding pink-purple tissue, which is the typical histological finding of rust spots (Fig. 3). The sign observed was therefore consistent with the phenomenon rust stain described in the literature.

### Ballistic considerations

From the evidence collected during scene investigation and autoptic examination, the man shot himself while lying in a supine position on his bed. He pulled the trigger with the first of his right hand while the other fingers were grasping the butt of the gun. The weapon was a Beretta Pistol Mod. 84F cal. 9 loaded with a 9 × 17 mm 9 Browning Corto (0.380 ACP) jacketed hollow-point bullet. The projectile used weighs 95 gr and can reach a muzzle velocity of 291 m/s, with a kinetic energy of 262 J. The morphology of the entrance wound was suggestive of a contact-range gunshot wound. The bullet path started from the frontal bone with an antero-posterior, slightly oblique, from right to left and from top to bottom course that damaged the left frontal, parietal, and occipital lobes.

The fact that the projectile was a low-velocity bullet with a relatively low kinetic energy, coupled to the documented substantial interaction with the cranial bones, led to the deformation and the retention of the bullet in the posterior cranial fossa. Most of the kinetic energy was dissipated during the impact with the fronto-ethmoidal and occipital pillars, so thick high-resistance regions of the frontal and occipital bones.

## Discussion

In case of firearm suicide, the determination of the manner of death is not always straightforward. The discrimination between homicide and suicide represents a challenge for the forensic pathologist when the circumstances are ambiguous [1]. Some evidence collected during the autoptic procedures are more common in suicides, such as the presence of a single, contact-range gunshot wound where the most affected anatomical districts are usually the temple, the oral cavity, and the frontal region [11]. The entrance wound is often surrounded by an abrasion collar and/or a contusion ring, while on the internal side, flecking with soot in the tissues can be seen [8, 12, 13].

Aside from the characteristics of the lesion caused by the firearm and the ballistic considerations, additional evidence can be complementary to the evaluation of the case. In the setting of firearm suicides, the weapon can leave several marks, signs, and traces on the person holding it that can guide the forensic pathologist in the investigation [14–16]. Occasionally, the weapon may remain in contact with the cadaver long enough to form a rust stain, which cannot be removed by rubbing [1, 17, 18].

Rust spots were first described as the result of tight contact between the skin and the gun in 1914, underlying how cataleptic *rigor mortis* was an absolute prerequisite for the formation of these marks [19]. To explain the phenomenon, Norton et al. hypothesized that the mechanism resided in the interaction between the iron of the weapon and the water and salt present in the sweat [20]. The peculiarity of this sign was further confirmed in 1995 by Bohnert et al., who reported a case of suicide committed with a rifle where the weapon left two ribbon-like traces of rust on the hand that remained in contact with the metal [21]. Also in this case, the formation of this mark was thought to be the result of prolonged contact between iron and moist skin. One of the most complete and in-depth studies on the biochemistry of rust stains is the excellent work of Ulrich and Zollinger in 2001 who tried to reproduce them on both the skin of the living and the dead in the experimental environment [10]. The tendency to produce rust stains of a greased gun was compared to a non-greased gun, on both wet and dry skin (Table 1).

**Table 1:** Results from Ulrich and Zollinger’s experiments [10]

Subjects	Skin–weapon features	Time for the formation of rust stain
Dead	Wet–greased	160 min
	Dry–greased	1320 min
	Wet–non-greased	135 min
	Dry–non-greased	280 min
Living	Wet–greased	78 min
	Dry–greased	170 min
	Wet–non-greased	27 min
	Dry–non-greased	130 min

Therefore, rust stains are not an exclusively *post mortem* phenomenon. As previously stated, the primary prerequisite for the formation of a rust mark on the skin is steady contact with the steel of the weapon. In real conditions, this is only possible in unconscious people. Another prerequisite is a minimum contact time between the weapon and the skin, which, according to Ulrich and Zollinger, was approximately 27 min for the living and 135 min for the dead [10]. In other studies, it was noted how variables such as skin pH, temperature, and others did not affect the time of formation of rust spots, while the amount of copper contained in the weapon was decisive [22, 23]. In particular, the lesser the amount of copper, the more difficult it would be for the rust stain to form.

The first histological examination performed by Norton et al. identified a brown-orange discoloration of the skin limited to the surface in contact with the weapon [20]. In 1996, Puschel et al. reproduced the phenomenon in an experimental setting using different weapons [24]. The extent of the produced metallization was confirmed by microscopic examinations: in the majority of cases, a thin layer of metallization of the stratum corneum extending towards the deep folds of the skin was detected with the Perls Prussian Blue staining, while migration of iron in the middle layers of the epidermis was observed in case of prolonged contact with very rusty weapons. Similarly, energy dispersion X-rays showed higher levels of iron in the rust-stained skin compared to that of the control [20].

Although the presence of rust stains on the skin demonstrates prolonged contact with the weapon, this is by no means an absolute proof that the affected hand fired the gunshot or that the shot was self-inflicted [25]. Some studies show how rust stains can coexist with other traces left by the weapon on the skin of the shooter, including residues and soot [26]. A peculiar characteristic of rust stains is that in addition to not being removable by rubbing, they sometimes reproduce exactly the metal medium that created them; in some cases, this feature has allowed to trace the weapon used in suicide even if this was no longer present at the crime scene [24]. Additionally, rust stains can also be produced by improvised weapons as illustrated by a case of suicide carried out by cattle stunners in which a large patch of rust was formed [27].

In the present case, the circumstances were strongly indicative of a suicide. The victim died as a result of a gunshot fired to the head which damaged the subject’s vital centers; moreover, the precise correlation between the rust mark and the design engraved on the gun’s trigger verified the compatibility of the weapon. Several studies described the finding of rust stains (Table 2), but this case report is particularly interesting not only for the rarity of the phenomenon but also for the precision with which the mark perfectly matches the grooves engraved on the trigger of the weapon found on the site of the event. Upon closer examination, the distance between the *striae* imprinted on the skin of the first finger was found to be equivalent to that of the longitudinal notches on the front part of the trigger (Fig. 2). Therefore, a rust stain with these features could represent crucial evidence for the identification of the murder weapon when this is not recovered during scene investigation. Aside from providing information about the characteristics of the trigger, the fact that a weapon has the potential to lead to the formation of this type of mark can give additional data on its composition, and possibly its maintenance. Furthermore, based on the aforementioned studies, rust stains could be useful to identify the weapon and also to evaluate the compatibility of the time in the medico-legal investigation.

**Table 2:** Part of the weapon found in contact with the skin and anatomical site of formation of the rust stain described in the literature

Authors	Type of weapon	Part of the weapon	Localization of rust stain
L. E. Norton et al. (1979) [6]	.308 Winchester rifle;	Gun barrel	Left palm
	.303 Enfield rifle	Gun barrel	Left palm
	.22 Caliber rifle	Gun barrel	Palm
M. Bohnert et al. (1995) [9]	Double barrel shotgun Rossi model. M-21 caliber 12/76	Gun barrel	Back of the right hand
K. Puschel et al. (1996) [10]	Tokarev TT-33 self-loading pistol	Pistol grip and gun barrel	Right palm; left forearm and left thigh
R. Pircher et al. (2017) [15]	Kerner–type stunner	Head piece; barrel	Left palm

## Conclusions

Rust stains represent a characteristic sign of firearm suicide in case of prolonged contact between the weapon and the skin, despite the fact that little is known about this phenomenon due to the limited literature on the subject. This sign can provide relevant information on the duration of the contact and, above all, on the nature of the weapon. For this reason, in cases of firearm suicide, along with the other well-documented findings, the external inspection should always aim towards the recognition of rust stains that suggest close and prolonged contact between the skin of the corpse with the weapon, in order to rule out the hypothesis of homicide with simulated suicide.

## Data Availability

N/A

## Code availability

N/A
